# Spatiotemporal analysis of lake chlorophyll-*a* with combined in situ and satellite data

**DOI:** 10.1007/s10661-023-11064-5

**Published:** 2023-03-14

**Authors:** K. Kallio, O. Malve, E. Siivola, M. Kervinen, S. Koponen, A. Lepistö, A. Lindfors, M. Laine

**Affiliations:** 1 Finnish Environment Institute, Helsinki, Finland; 2 Luode Consulting Ltd, Espoo, Finland; 3 Finnish Meteorological Institute, Helsinki, Finland

**Keywords:** Chlorophyll-*a*, Water framework directive, Spatiotemporal interpolation, Satellite images, Automated measurements, Lake

## Abstract

We estimated chlorophyll-*a* (Chl-a) concentration using various combinations of routine sampling, automatic station measurements, and MERIS satellite images. Our study site was the northern part of the large, shallow, mesotrophic Lake Pyhäjärvi located in southwestern Finland. Various combinations of measurements were interpolated spatiotemporally using a data fusion system (DFS) based on an ensemble Kalman filter and smoother algorithms. The estimated concentrations together with corresponding 68% confidence intervals are presented as time series at routine sampling and automated stations, as maps and as mean values over the EU Water Framework Directive monitoring period, to evaluate the efficiency of various monitoring methods. The mean Chl-a calculated with DFS in June–September was 6.5–7.5 µg/l, depending on the observations used as input. At the routine monitoring station where grab samples were used, the average uncertainty (standard deviation, SD) decreased from 2.7 to 1.6 µg/l when EO data were also included in the estimation. At the automatic station, located 0.9 km from the routine monitoring site, the SD was 0.7 µg/l. The SD of spatial mean concentration decreased from 6.7 to 2.9 µg/l when satellite observations were included in June–September, in addition to in situ monitoring data. This demonstrates the high value of the information derived from satellite observations. The conclusion is that the confidence of Chl-a monitoring could be increased by deploying spatially extensive measurements in the form of satellite imaging or transects conducted with flow-through sensors installed on a boat and spatiotemporal interpolation of the multisource data.

## Introduction

Monitoring of water quality of lakes has conventionally been carried out using manually acquired, grab water samples analysed in the laboratory. Due to limited resources, monitoring is often limited to one station typically located at lake deep, with the assumption that this station represents the whole lake. The number of water samples per year in routine monitoring is typically low, and therefore, phytoplankton blooms can be missed. The EU Water Framework Directive (WFD) recommends a sampling frequency of twice per year for phytoplankton (Birk et al., 2012), but some lakes are monitored more frequently, for example, once or twice a month. Even more frequent sampling may lead to information gaps due to the occurrence of processes with a shorter temporal scale than the sampling frequency (Dubelaar et al., 2004). Moreover, it is necessary to consider sample collection and handling method accuracy, the effect of laboratory methodology error, as well as how representative the data are for the objective of the study (EPA, 2002).

The temporal and spatial deficiencies of conventional monitoring can be reduced by using automatic sensors and satellite remote sensing (Earth Observation, EO). The automatic sensor measurements in lakes have been used to understand and predict the state of globally selected lakes (The Global Lake Ecological Observatory Network GLEON, http://gleon.org/, Rose et al., 2016). Furthermore, they can be used in lake management to estimate the eutrophic status of lakes in determining the environmental actions and legislation needed. Chlorophyll-*a* (Chl-a) concentration is commonly used as a proxy for phytoplankton biomass and can be estimated by continuous fluorometer measurements. In the WFD, high frequency of Chl-a measurements are valuable for defining the required frequency of conventional monitoring so that the confidence levels for status assessment are high enough to estimate Chl-a in lakes with highly varying phytoplankton and in lakes which fluctuate between good and moderate ecological status (Marcé et al., 2016). However, automatic sensor measurements are costly, and high-quality estimations require continuous maintenance of the sensors.

The availability of satellite images for lake monitoring has increased in recent years, particularly due to the European Copernicus Sentinel (https://sentinels.copernicus.eu) and NASA/USGS’s Landsat (https://landsat.gsfc.nasa.gov/) programs. The spatial resolution of satellite images suitable for water quality estimations varies typically between 20 and 300 m. The EO-based Chl-a products have been used in estimating the status of WFD waterbodies in lakes (Ansper & Alikas, 2019; Bresciani et al., 2011; Markogianni et al., 2022; Papathanaopoulou et al., 2019) and coastal waters (Attila et al., 2018; Gohin et al., 2008).

The advanced monitoring techniques bring additional information on temporal and spatial variation, but to optimize their joint use in conjunction with conventional methods requires proper tools. In spatiotemporal statistical interpolation, multisource observations are harmonized and interpolated to fill in data gaps and provide an overview of the variation and uncertainty of the estimated variables. Spatial interpolation provides combined spatial concentration estimates for the measurement days and has been used, e.g. in Chl-a estimation in coastal waters (Pulliainen et al., 2004) and the lake environment (Wilkie et al., 2015). Spatiotemporal interpolation also provides spatial estimates for the periods lacking observation data and has been applied to lakes, for instance, to estimate harmful algal bloom probabilities (Fang et al., 2019) and to assess dissolved inorganic nitrogen (Wang et al., 2019). The spatiotemporal algorithms used in water quality applications have varied (Gunia et al., 2022). Observations on the application of geostatistical methods have typically included two of three of the following information sources: (i) conventional water sampling, (ii) automatic station measurements either stationary or from a moving ship, and (iii) EO images.

The objective of this study was to compare the spatiotemporal variability and uncertainty in the estimated Chl-a concentrations of various combinations of observations. The study was conducted in the mesotrophic Lake Pyhäjärvi in Southwest Finland in 2009, and observations consisted of routine monitoring (grab water samples), automatic lake station sampling, and satellite measurements. Interpolation was done using a data fusion system based on an ensemble Kalman smoother algorithm. First, we present the interpolated Chl-a and its confidence intervals with various measurement combinations at routine and automatic measurement stations. Second, spatial variation of interpolated Chl-a is illustrated on maps. The mean concentration and its uncertainty at selected locations and areas are compared using various measurement combinations. We show that EO data decrease the uncertainty of Chl-a considerably when the whole research area and WFD monitoring season are considered.

## Description of Lake Pyhäjärvi

**Fig. 1:**
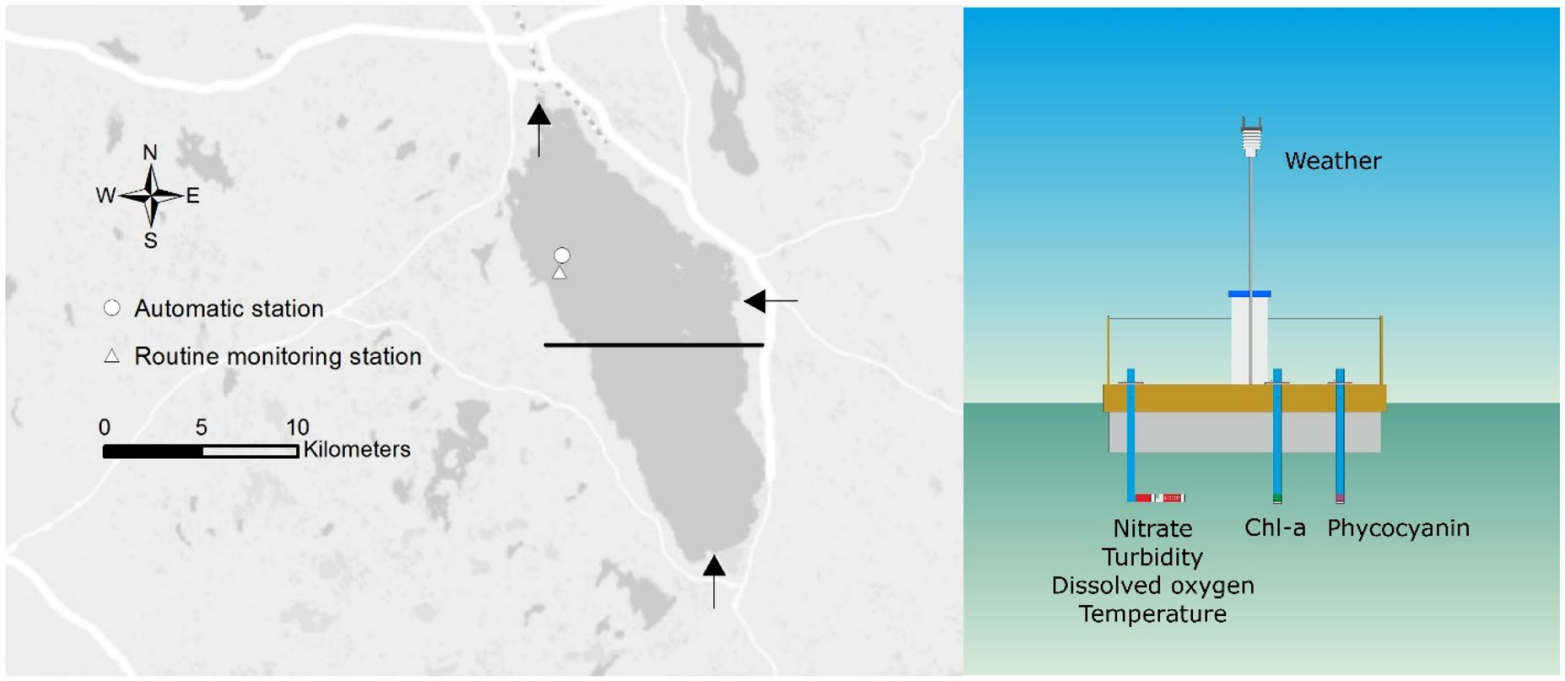
Lake Pyhäjärvi with the locations of the routine monitoring and automated station (left panel) and the structure of the automated station (right panel, see text for details). The black line on the map indicates the northern part of the lake, on which we focus in this study. The River Yläneenjoki (southern shore) and River Pyhäjoki (eastern shore) inflows, as well as the River Eurajoki outflow (northern shore), are indicated by arrows. Background map: © National Land Survey of Finland, licence no. 7/MYY/06

Pyhäjärvi is a large (155 km^2^), shallow mesotrophic lake in Southwest Finland (Fig. 1) with a mean depth of 5.5 m, the deepest point of which is 26 m (coordinates 61.0256° N, 22.2051° E). Its ecological status has been good to moderate during the WFD era, which began in the early 2000s. Hydrological and hydrobiological research on the lake has been ongoing for decades, and it is one of the most studied lakes in Finland. Its water chemistry and plankton species composition have been monitored from the 1960s to the present. Nutrient and plankton community monitoring efforts intensified starting in 1980, and since then, the state of the lake has been summarized by Ventelä et al. (2007) and Ventelä et al. (2016). Lake Pyhäjärvi is a highly valuable lake in terms of water supply and recreational use. It also has exceptionally high fish productivity (Sarvala et al., 1998) making it an important lake for commercial fishing.

Two major rivers, Yläneenjoki (233 km^2^) and Pyhäjoki (78 km^2^), discharge into the lake (Fig. 1). Agriculture in the catchment area consists mainly of cereal production and poultry husbandry (Pyykkönen et al., 2004; Rankinen et al., 2021). The Yläneenjoki catchment is responsible for 53–57% of the external phosphorus load into Pyhäjärvi, while Pyhäjoki is responsible for 10–12% (Ventelä et al., 2007). Increased eutrophication became a major concern in the late 1980s: between 1970 and 1992, the nitrogen concentration in the lake increased by 30% (Ekholm et al., 1997), and the phosphorous concentration doubled, but the nutrient concentrations later decreased (Ventelä et al., 2007; Ventelä et al., 2016).

Lake Pyhäjärvi has served as the Finnish flagship area of several national and EU projects, such as CatchLake (Lepistö & Huttula, 2008), REFRESH (Lepistö et al., 2013), and GISBLOOM (Malve et al., 2016). It is one of the most important pilot study lakes in Finland where catchment and lake modelling (Huttula, 1994; Lepistö et al., 2013; Malve et al., 2007; Rankinen et al., 2021), automatic sensor measurements, transect measurements from a moving boat (Lepistö et al., 2010), as well as satellite remote sensing (Kallio, 2012; Lepistö et al., 2010) and data assimilation (Mano et al., 2015) techniques have been applied. The results of the Pyhäjärvi automated station (Fig. 1), described in detail below, have been used in such cases as the study of heat loss from lakes (Woolway et al., 2018) and the impact of episodic weather-induced events on lakes (Kuha et al., 2016).

## Material and methods

### Water sampling and laboratory determination methods

Lake Pyhäjärvi was routinely monitored at the lake deep by grab water samples and laboratory analyses once or twice a month during May–September in 2009. Chl-a samples were taken as composite samples from a depth of 0–2 m. In addition, nine water samples were taken during June–October at the automated station from a sensor depth of 1 m, for the calibration of the sensor measurements. The concentration of Chl-a was measured in the laboratory with a spectrophotometer after extraction with hot ethanol (ISO 10260, 1992, GF/C filter). Phytoplankton samples (composite 0–2 m) taken at the routine monitoring station were preserved with acidic Lugol’s solution. Cells were enumerated by microscopy, and cell counts were converted to biovolumes as described in Räsänen et al. (2006).

### Automatic station and sensor corrections

The Pyhäjärvi lake automatic station was founded close to the routine monitoring station (900 m apart, see Fig. 1) in August 2008. The station was constructed of a float (3 m × 3 m × 0.5 m) with four plastic pontoons and a rectangular wooden deck. The float with a steel case for dataloggers and other equipment was anchored to the lake bottom from each corner. The depth at the location was approx. 20 m.

Chl-a and phycocyanin fluorescence were measured with Trios’s Micro Flu fluorometer (470-nm excitation and 685-nm emission wavelengths) and Micro Flu blue fluorometer (620-nm excitation and 655-nm emission wavelengths). The phycocyanin fluorescence measurements were utilized in the correction equation of the Chl-a fluorometer (see later). Both sensors were suspended on the side of the float at a depth of 1 m. Other measured variables, not utilized in this study, were turbidity, nitrate and dissolved oxygen concentrations, and water temperature.

Dataloggers, other electronics, a compressed air tank, and batteries (two 60-Ah units) were mounted inside a steel/aluminium case (0.4 × 0.8 × 1.4 m). A Vaisala 500 series weather station/transmitter was installed on the top of the case (about 2 m above the water level). Data from the station were transmitted via GSM network to Luode Consulting Ltd.’s database. The optical sensors were kept clean with compressed air, which was taken from 10-l 200 bar divers’ tanks. The cleaning cycle was controlled by dataloggers. In addition, all sensors were manually cleaned during service visits, typically once a week.

Before the measurement season, the Chl-a and phycocyanin fluorometers were calibrated with ultrapure water. The fluorescence measurements were transformed to Chl-a during the measurement season using coefficients based on data from other Finnish lakes where the same fluorometer models have been used. The phycocyanin fluorometer readings were first converted to wet weight of cyanobacteria with the empirical coefficients obtained from fluorometer and cyanobacteria biomass measurements at Lake Hiidenvesi, which is located in Southern Finland. The cyanobacteria biomass measurements were fine-tuned using biomass measurements (microscopic counting) at the routine measurement station after the measurement season by multiplying the result with 0.41 (R^2^ = 0.94,* N* = 7, standard deviation SD = 0.13 mg/l, range 0.04–0.86 mg/l). The final calibration of Chl-a was made after the measurement season based on the Chl-a concentration measured at the automated station and the routine monitoring station. All Chl-a laboratory determinations were made in the Environmental Laboratory of the Water Protection Association of river Kokemäenjoki.

**Fig. 2:**
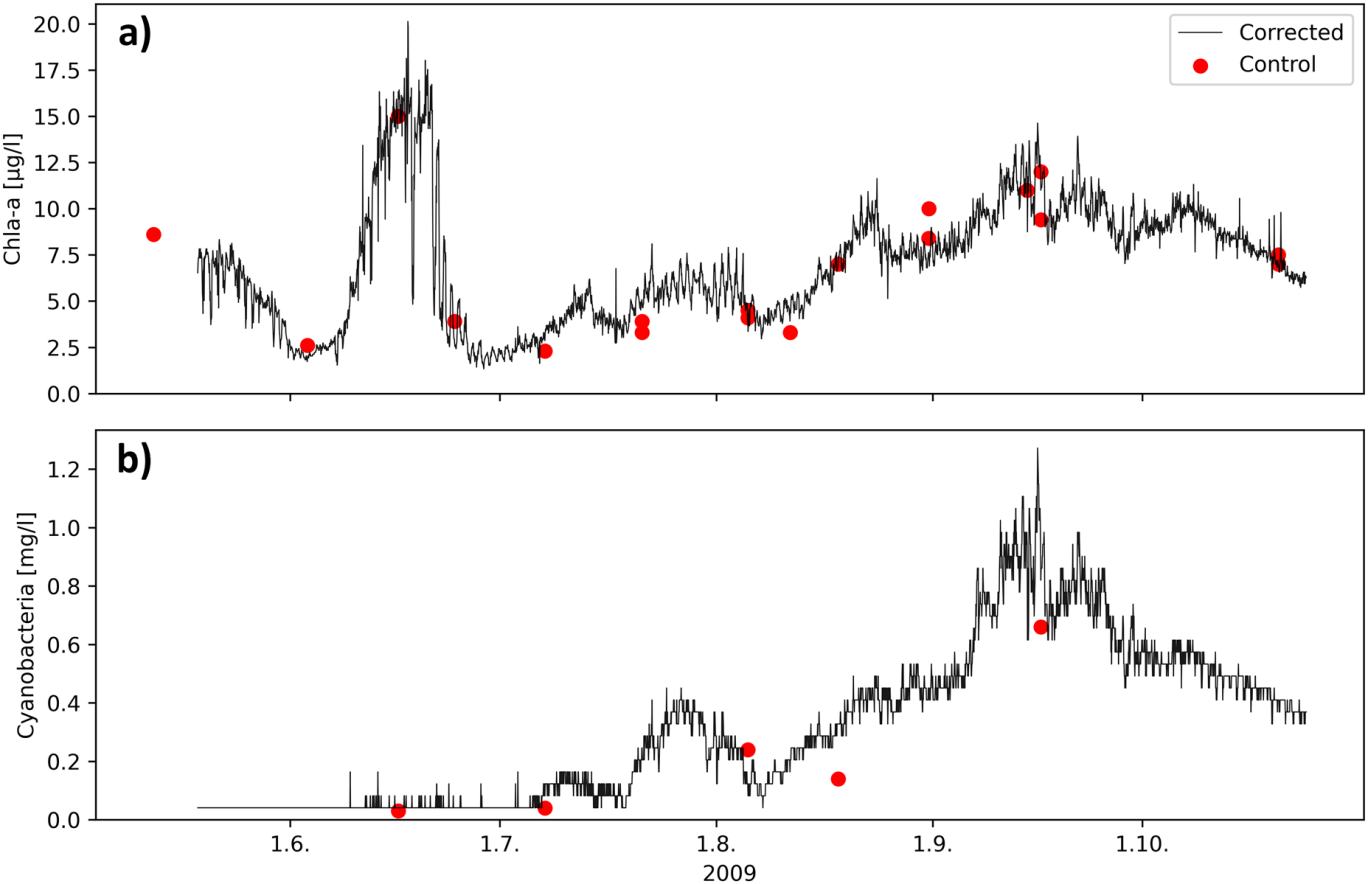
Corrected Chl-a concentration (**a**) and cyanobacteria biomass (**b**) measured with fluorometers (hourly data) at the Pyhäjärvi automated station in 2009, together with control samples measured in the laboratory

The fluorescence/Chl-a ratio usually varies according to the phytoplankton species. In Lake Pyhäjärvi, this ratio was clearly lower in the late summer rather than in the early summer. In cyanobacteria most of the Chl-a is located in the non-fluorescing photosystem I, which leads to low Chl-a-specific fluorescence, when compared to eukaryotic algae (Campbell et al., 1998). The late summer phytoplankton in Lake Pyhäjärvi is usually characterized by cyanobacteria, which was confirmed in 2009 by microscopic counting. Therefore, we made the final calibration using multiple regression with Chl-a and cyanobacteria biomass as independent variables: Chl-a_corrected_ = Chl-a_sensor_ + 3.3*CB_sensor_—0.8 (R^2^ = 0.93, *N* = 19, SD = 1.1 µg/l, relative SD = 16%), where CB is cyanobacteria biomass (wet weight), a and b are empirical coefficients, and relative SD is calculated from the SD and the mean water sample Chl-a. The same type of correction with cyanobacteria as an additional variable has been applied to Baltic Sea data by Seppälä et al. (2007) and Lake Vesijärvi data in Finland (Anttila et al., 2012). The corrected time series of Chl-a and cyanobacteria are presented in Fig. 2. During the Chl-a peak in June, phytoplankton was dominated by diatoms.

The fluorescence yield of phytoplankton can be considerably lower in the middle of the day during clear sky conditions than at night due to light-induced fluorescence quenching (e.g. Rousso et al., 2021). This phenomenon was also observed at Lake Pyhäjärvi when hourly fluorometer measurements were compared to the intensive global radiation measurement of a nearby weather station. Therefore, we used 9 o’clock (local time) automatic station fluorometer measurements in the spatiotemporal calculation. The possible light-induced fluorescence quenching is negligible at this time, and in addition, the control measurements were usually taken around 9 o’clock.

### EO data and calibration

The satellite-based Chl-a data were generated from Envisat MERIS images (spatial resolution 300 m, https://earth.esa.int/eogateway/instruments/meris) provided by the European Space Agency (ESA). The following processing steps were applied to the Level 1b datasets from the years 2009 and 2011, after selecting the non-cloudy images:

1. Radiometry correction—This step performs the basic corrections (calibration, smile correction, and equalization) that ensure the data quality for the next steps.
2. ICOL 2.10 (Snapshot) adjacency correction processor—The processor reduces the effects of nearby land areas on the radiance data observed over water.
3. FUB/WeW 1.2.8 water quality processor (Schroeder et al., 2007)—This step converts the radiance values in the Level 1b datasets into concentrations of water quality parameters (including Chl-a) with the use of neural networks. In addition, the processor provides various flags that indicate the quality and usability of the pixel values.
4. Georeferencing into the Finnish coordinate system—The observations made in satellite geometry are matched with ground coordinates so it is possible to compare in situ and EO values from the same location. The processing was done using the BEAM 4.10.3 software package (http://www.brockmann-consult.de/cms/web/beam/).

After processing the pixel Chl-a, related flag values were extracted from the location of the automatic station using a 3 by 3-pixel window. The means of Chl-a concentrations within the windows were then compared with the same-day fluorometer Chl-a measurements from the automated station (see “Automatic station and sensor corrections” section for details). The comparison showed that the FUB processor output (Chl-a_Satellite_) overestimated the Chl-a concentrations. Therefore, the following calibration equation for corrected Chl-a (Chl-a_Calib_) was then formed:

1 $$\mathrm{Chl-a}_\mathrm{Calib} = 0.346^* \;\mathrm{Chl-a}_\mathrm{Satellite} + 3.76$$

After applying the calibration equation, the R^2^ was 0.658, and SD was 2.56 µg/l (*N* = 15). Finally, the calibration equation was used to generate the raster maps of Chl-a for eight cloudless days in 2009.

### Data fusion

#### Algorithm

The calculations in the data fusion system are based on an ensemble Kalman filter and smoother algorithms. We will present the basic mathematics here, but an interested reader may refer to Gunia et al. (2022) for more details. The basic description starts with the following state space equations:

2 $$\begin{array}{c}{{\varvec{x}}}_{k}={M}_{k}\left({{\varvec{x}}}_{k-1},{\varvec{\theta}}\right)+{{\varvec{\eta}}}_{k}\\ {{\varvec{y}}}_{k}={H}_{k}\left({{\varvec{x}}}_{k},{\varvec{\theta}}\right)+{{\varvec{\varepsilon}}}_{k}.\end{array}$$

These two equations describe the evolution of the multi-dimensional state, ${{\varvec{x}}}_{k}$ (the true Chl-a concentrations) at time indexed with $k$. Observation vector ${{\varvec{y}}}_{k}$ contain all (noisy) measurements at time $k$. The parameters of the model are stacked as **θ**, the evolution of the state is described with the function ${M}_{k}(\cdot ,\cdot)$, and the connection between the measurements and the true state is described by the function ${H}_{k}(\cdot ,\cdot )$. Both the evolution of the state and the measurements contain stochastic components ${{\varvec{\eta}}}_{k}$ and ${{\varvec{\varepsilon}}}_{k}$. In the algorithm used here, the evolution of the model is given as ${M}_{k}\left({{\varvec{x}}}_{k-1},{\varvec{\theta}}\right)=\alpha \left({\varvec{x}}-{\mu }_{b}\right)+{\mu }_{b}$ where α controls the speed of how fast the state returns to the mean value ${\mu }_{b}$. In addition, with suitably pre-processed observations, we can assume ${H}_{k}\left({{\varvec{x}}}_{k},{\varvec{\theta}}\right)= {{\varvec{x}}}_{k}$, so that there are no transformations needed between the observations and the state.

The stochastic components ${{\varvec{\eta}}}_{k}$ and ${{\varvec{\varepsilon}}}_{k}$ are assumed to follow multivariate normal distributions, where the means are zeros and correlation matrices ${\varvec{Q}}$ (for state uncertainty) and ${\varvec{R}}$ (for measurement uncertainty) are defined as follows. The current system uses an isotropic spherical correlation structure

3 $${Q}_{ij}=\left\{{\sigma }_{s}^{2}\begin{array}{cc}\left[1-\left(\frac{3}{2}\frac{{h}_{ij}}{l}-\frac{1}{2}{\left(\frac{{h}_{ij}}{l}\right)}^{3}\right)\right],& \mathrm{if}\;{h}_{ij}<l\\ 0,& \mathrm{otherwise},\end{array}\right.$$

where ${h}_{ij}$ is the distance between the locations where the states (true Chl-a) are estimated, ${\sigma }_{s}^{2}$ is a parameter that controls the level of overall uncertainty, and $l$ is a parameter that controls how state values which are far from each other affect one another (Gelfand et. al., 2010). The correlation matrix associated with the measurements is defined as a diagonal matrix

4 $${{\varvec{R}}}_{ij}=\left\{\begin{array}{cc}{\sigma }_{s}^{2},& if\; i=j\\ 0,& \mathrm{otherwise}\end{array}\right.$$

where ${\sigma }_{o}^{2}$ is a parameter that defines the uncertainty of the observations.

When the operators ${M}_{k}$ and ${H}_{k}$ are linear, as in our case, the uncertainties are Gaussian, and we assume certain Markovian dependence structures. The estimation of the state can be done with a classical Kalman filter and smoother algorithms. For each time $k$, the Kalman filter algorithm estimates the current state by considering the measurements of the current and past states. For spatiotemporal interpolation performed in the data fusion system, we use the Kalman smoother, which, in addition to the past observation, uses future observations when inferring the state of each time point. The size of the modelled region can be large, which makes the system computationally demanding. This is due to the storage and computational requirements when the model domain is large. For large spatial domains, the usual remedy is to use an ensemble-based Kalman filter and smoother, where the system state ${{\varvec{x}}}_{k}$ and error ${\varepsilon }_{k}$ are represented by an ensemble (or collection) of possible realizations of the states and errors. In other words, the ensemble Kalman filter is a Monte Carlo approximation of the exact Kalman filter. The size of the chosen realizations in the ensemble is usually small compared to the number of states. In our experiments, we used 500 as the ensemble size.

For computational reasons, data (i.e. measurements) are often transformed before modelling with the Kalman filtering (or smoothing) approach. Here we took the logarithm of the data and model instead of the actual data. This is done to make positive data (Chl-a measurements) remain strictly positive when modelled. If this transformation was not done, physically invalid predictions may arise (i.e. negative Chl-a concentrations).

#### Data fusion parameters

The data fusion algorithm is configured using parameters to be explained later in this section. The goal of this section is to provide an intuitive understanding of each of these parameters. For a detailed mathematical description of the model parameters, please refer to the previous section or Gunia et al. (2022).

All measured Chl-a concentrations (and estimated Chl-a) are tide to 60 m × 60 m cells, which are generated for the entire Pyhäjärvi lake. The final model domain was defined by removing a 500-m inward buffer from high-accuracy (10 m) lake shoreline data. The domain reduction was done to exclude potentially inaccurate shallow water EO observations near the shoreline. Before using the algorithms, all data are transformed via natural logarithm.

The data fusion system interpolates observations in space and time onto a regular grid. The background value of Chl-a concentration is set to 6.2 µg/l (${\mu }_{b}$, background mean), which is the median Chl-a concentration of routine monitoring measurements from June to September in 2004–2008 (computed from 25 measurements). In the absence of observations from the surrounding area, the predictions are drawn upon this value allowing them to coincide with past measurements. Similarly, modelling uncertainty of Chl-a concentrations (${\sigma }_{s}$) in the absence of data is aligned using the standard deviation of past manual measurements. The upper bound confidence interval (68%) of the data fusion estimates are made to match the maximum value of past routine monitoring samples (from years 2004–2008), which is 20 µg/l. In the absence of data, the predicted uncertainty coincides approximately with the overall variability of past measurements.

Measurement error standard deviations ( ${\sigma }_{o}$) are 10% for routine monitoring, 16% for automatic measurements, and 34% for EO. The routine monitoring error was estimated in the water laboratory that conducts the determinations and was available in the VESLA database. The other two errors were estimated by comparing the sensor estimations to in situ measurements (see “Automatic station and sensor corrections” section).

The correlation length, $l$, in the data fusion model is set to 8 km. The correlation length was selected by fitting a spherical variogram model to the EO data to describe its spatial continuity and by finding the length after which the values are not correlated. The temporal correlation (autocorrelation) of the EO data for Lake Pyhäjärvi is 3 days, which means that observations at the same location do not correlate after this. Model drift ($\alpha$) is set to 0.97, which equals the mean estimate returning to the background mean in 3 days in the absence of any data. This behaviour equals the temporal correlation of Lake Pyhäjärvi.

#### Data fusion system

The data fusion system (DFS) is operated on SYKE’s premises on a physical server with Xeon E5-2667@3.2 GHz and 64 GB RAM that runs Windows Server 2016. The server hosts both the system database (PostgreSQL 9.5.13) and computational software (Python 3.6.9). Satellite data products are harmonized with the system directly from SYKE’s open access WCS-interface and with in situ data from the ODATA interface. As a result of harmonization, all observations and their uncertainties are presented as numerical values in the pre-defined discrete spatiotemporal grid. Data fusion is operated through command line programs, and the results can be analysed using QGIS 3.4.2. The computational core is implemented as a general-purpose library called EnDAS (ensemble data assimilation system), and its source code is available under an open source licence (Gunia, 2018).

## Results

### Chl-a time series

We studied the temporal variation of interpolated Chl-a concentrations and its uncertainty using standard deviation SD (68% confidence interval) at routine monitoring and automated stations (Fig. 1). The routine monitoring station is the official monitoring site of WFD, with water sampling once or twice a month, while at the automated station, Chl-a concentrations were available daily.

**Fig. 3:**
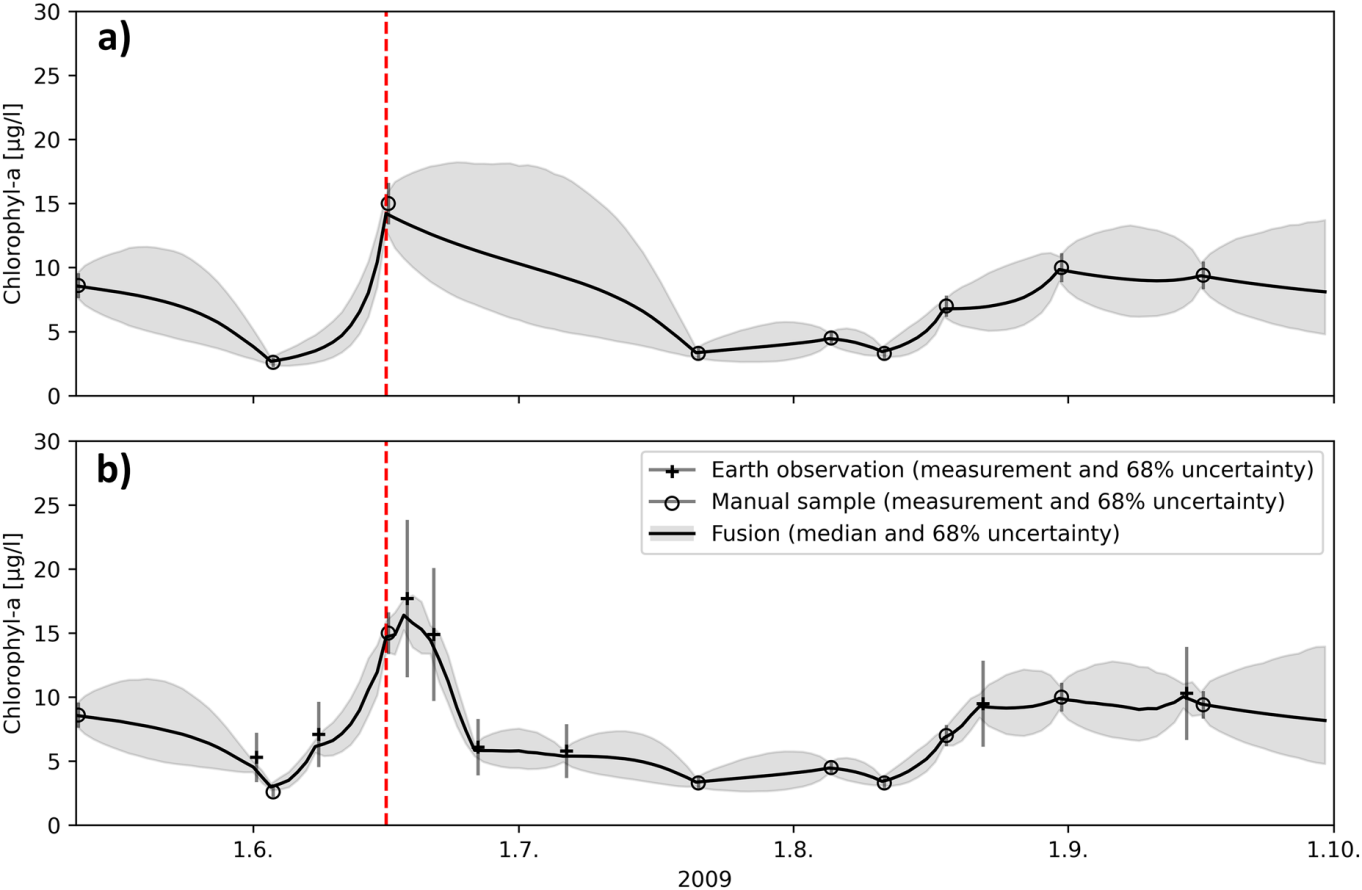
Chl-a time series calculated by DFS at the routine monitoring station with **a** routine monitoring station measurements and **b** routine monitoring station and EO measurements as input

Time series plots are useful in demonstrating how individual observations affect estimation uncertainties. At the routine monitoring station, the confidence interval of the DFS is wider the longer the time gap between two consecutive routine measurements (Fig. 3a). Maximum SD was reached at the beginning of July when the time between consecutive measurements was almost 5 weeks (Fig. 1a). The uncertainty is presented at a 68% confidence interval, which can be interpreted as a central posterior interval that contains the true values at a 68% probability. The interval is not symmetric since computations were done using a logarithmic scale. On a logarithmic scale, the interval is equal to the mean ± one standard deviation.

By adding the EO observations to the DFS, we get more realistic Chl-a concentrations in the periods with no routine monitoring results (Fig. 3b). The impact is particularly clear in late June–early July when EO observations provide information on the decline of the Chl-a peak in the middle of June. The occasional EO observations also reduce the uncertainty, particularly when they occur during periods when routine water samples were unavailable.

**Fig. 4:**
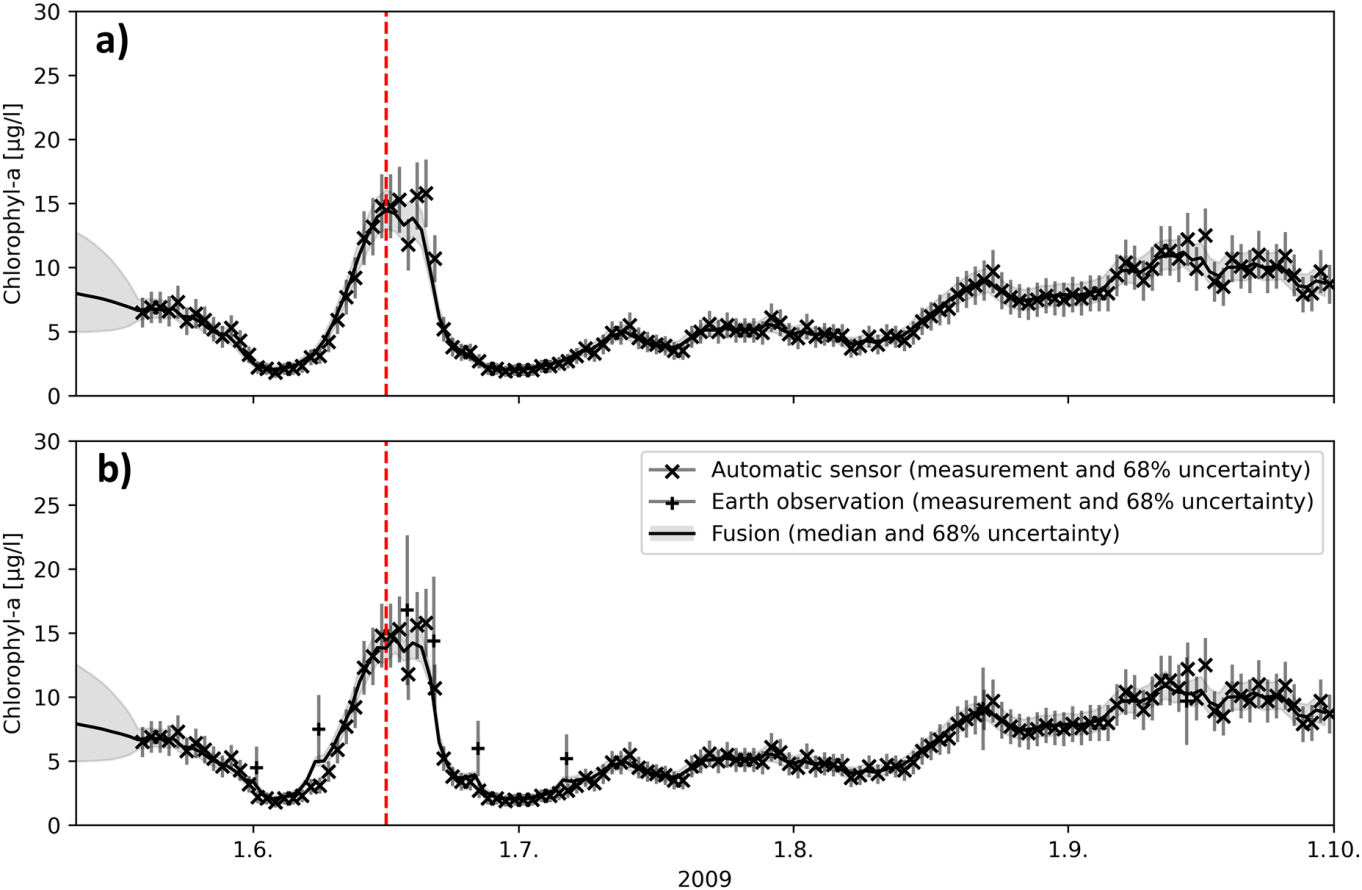
Chl-a time series calculated by DFS at the automated station with **a** automated station and routine monitoring station measurements and **b** automated station, routine monitoring station, and EO measurements as input

At the automated station, the confidence interval remains low because of the availability of daily measurements (Fig. 4a). The routine sample measurements at the lake deep are included in the DFS run in Fig. 4a**,** but they have no notable impact on the DFS result because of the distance (0.9 km) between the stations and low measurement frequency. Including EO observations in the DFS run has very little impact on the Chl-a (Fig. 4b). A small peak in the DFS result can be seen in the EO observation on the 8th of June.

The WFD ecological classification of Lake Pyhäjärvi is based on the mean concentration of Chl-a measurements taken from the routine monitoring station in June–September, while the automated station and EO provide a lot of additional information. To distinguish the difference, we compared the mean Chl-a concentration and confidence interval calculated by DFS with various data combinations from the routine monitoring station and the automated station located nearby in June–September.

Mean Chl-a concentrations were quite similar amongst different data combinations, though slightly higher at the routine monitoring station (Table 1). One of the samples taken at the routine stations (June 16, 2009) happened to be close to the Chl-a peak, but the sampling interval used (2–4 weeks) may miss phytoplankton periods. The duration of the Chl-a peak in June, for example, was only 2–3 weeks.

**Table 1:** Mean Chl-a concentration and SD at the routine monitoring and automated stations in June–September 2009. The results are calculated from daily DFS results with various data combinations

Location	Data in DFS	Mean Chl-a µg/l	SD µg/l	Relative SD* %
Routine monitoring station	Routine sampling	7.32	2.67	34.6
	Routine sampling and EO	7.10	1.63	23.7
Automated station	Automated station and routine sampling	6.56	0.70	10.7
	Automated station, routine sampling, and EO	6.64	0.65	9.9

^*^Calculated from daily Chl-a and SD

Automatic data markedly reduces the SD (from 34.6 to 10.7%) compared to the routine monitoring data (Table 1). The inclusion of EO data decreased the SD from 34.6 to 23.7% at the routine monitoring station, but its effect on the automated station, with daily sensor data, was very low. In DFS, which utilizes all types of available data, the Chl-a concentration at each station was not only influenced by EO Chl-a pixel-derived concentrations from the automated station, but also by satellite image pixel-derived concentrations collected from the nearby area. Here, the SD of DFS was approximated by dividing the 68% confidence interval by two. This allows for the comparison of DFS SD with the SDs of normal distributions as the SD of the normal distribution is also equal to half the confidence interval. For the automated station, the SD is less than the SD of the automatic measurements when only automatic measurements are available (10.7% relative SD of DFS vs 16% relative SD of the automatic measurements). The automated station’s measurements are collected daily, which reduces the estimated SD as the autocorrelation length of Chl-a is 3 days.

Measurement and estimation uncertainties are seldom included, e.g. in the WFD reporting. The accredited water laboratories report uncertainties for their determination methods, which can be added to the WFD status reports. At the routine monitoring station, DFS estimates the SD for the whole WFD period (Fig. 3a). DFS represents the overall uncertainty of routine monitoring better than laboratory error alone as it considers the length of periods without routine samples. The longer the period, the higher the error becomes.

Through the inclusion of EO data in the DFS, in addition to routine monitoring data, we get shorter periods containing no data. Although the uncertainty of EO is higher than that of routine measurements (due to the number of samples available daily when EO data are available), the overall DFS uncertainty at the routine monitoring station is lower than that of DFS interpolation with routine monitoring measurements only.

### Spatial Chl-a

The DFS calculates the daily spatial distribution of Chl-a in a 60 m × 60 m grid, which can be used to identify the differences between the various observation combinations and the resulting uncertainty across the study area. Here, we first present the results for the 16th of June 2009 using (1) all observations and (2) manual and automatic measurements only. The date selected was a date on which samples were taken both at the routine monitoring station and the automated station. The next EO observations were from samples taken on the 18th and 21st of June, and the previous were taken on the 8th of June.

**Fig. 5:**
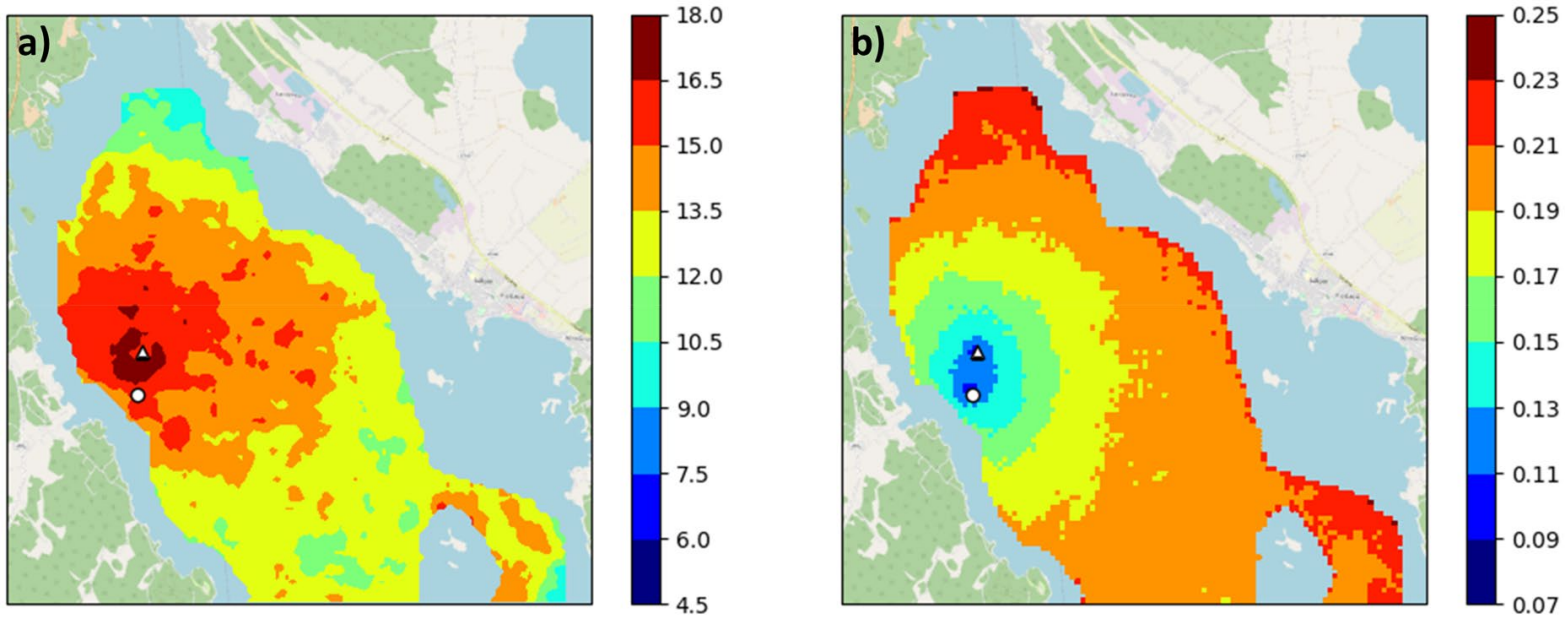
DFS calculated Chl-a concentration (µg/l) **a** and relative SD (SD/Chl-a ratio) **b** on the 16th of June 2009 with all data (routine sampling, automated station, and EO) as input. The routine sampling station is indicated by a circle and the automated station by a triangle

The interpolated Chl-a at the routine monitoring station on the 16th of June 2009 was 15.3 µg/l when all observations were used as DFS input. Chl-a decreased with increasing distance from the area around both stations with the lowest Chl-a (about 10 µg/l) concentration occurring in the north (Fig. 5a). Correspondingly, SD was lowest near both stations (about 11% for both) and highest (about 22%) in the northern and eastern parts of the study area (Fig. 5b).

**Fig. 6:**
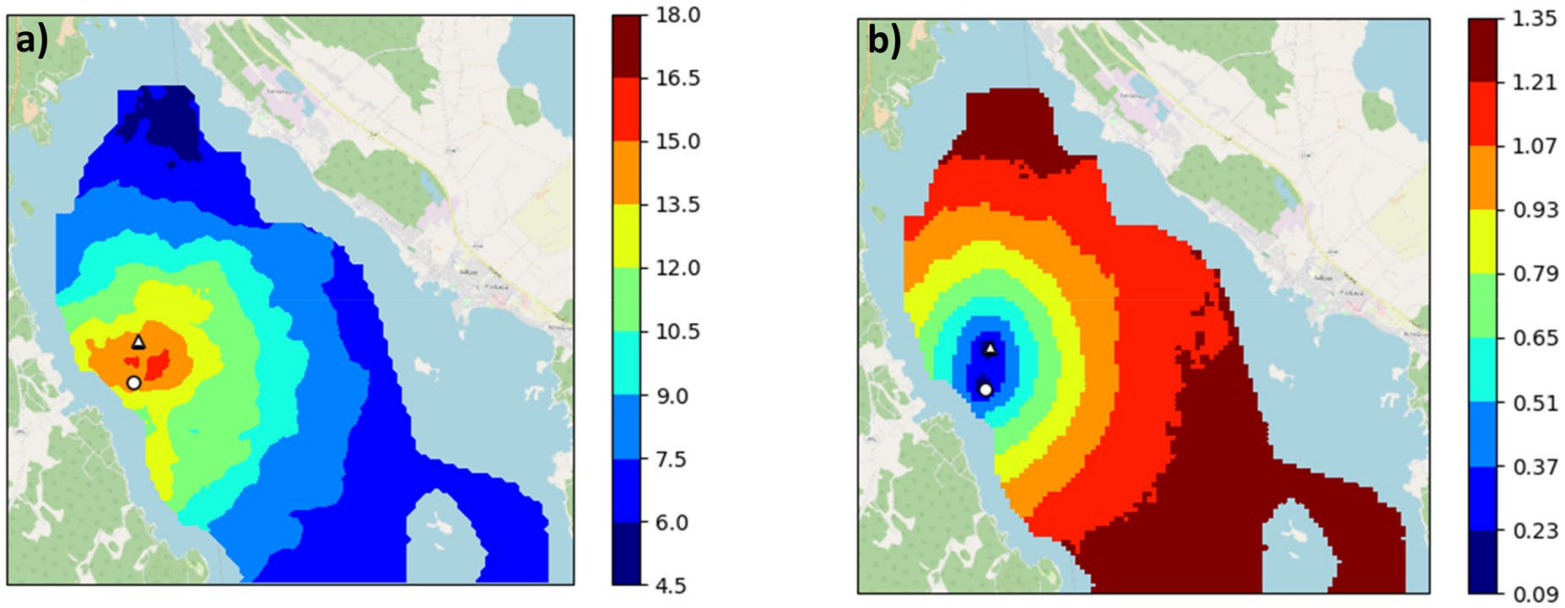
The DFS calculated Chl-a concentration (µg/l) **a** and relative SD (SD/Chl-a ratio) **b** on the 16th of June 2009 with routine sampling and automated station data as input. The routine sampling station is indicated by a circle and the automated station by a triangle. Note that the scale for SD in **b** is different to that of Fig. 5b

Interpolated Chl-a concentrations for the case without EO data (Fig. 6) were 14.9 µg/l at the routine monitoring station and lowered smoothly to 6–7 µg/l in the northern and south-eastern parts (Fig. 6a). The SD increased dramatically from 23% at the routine station up to 120–130% (Fig. 6b). Figure 5 looks noisier than Fig. 6 due to EO noise being locally correlated, which also affects the fusion result. Figure 6 is generated with more data, which gives a smoother outcome.

Based on a comparison of spatial results, the EO data decreased the uncertainty of interpolation substantially throughout the northern part of the lake. Without them interpolated Chl-a concentrations were averaged based on the given background mean concentration (6.2 µg/l), resulting in higher SDs in the Chl-a concentrations observed at locations far from the stations.

To compare the results for the WFD observation period, we integrated the daily spatial DFS results from June–September by calculating the mean and SD of Chl-a concentrations.

The mean Chl-a concentration of the June–September DFS simulations and the routine monitoring measurements with and without automated station measurements varied only slightly (6.47 and 6.33 µg/l, Table 2). The mean Chl-a concentration was about 1 µg/l higher when EO data were included in the simulations. However, the SD was lower when EO data were included (approx. 3 µg/l vs approx. 7 µg/l, Table 2) which in turn decreases the uncertainty of the status estimation. The use of daily automatic data in addition to the routine station data decreased the spatial error only slightly.

**Table 2:** Mean Chl-a concentration and error in the northern part of Lake Pyhäjärvi in June–September 2009. The results were calculated from the daily DFS maps of Chl-a with various data combinations

Data used in DFS	Mean Chl-a µg/l	SD µg/l	Relative SD* %
Routine station	6.47	7.22	112.1
Routine station and EO	7.33	2.97	43.6
Routine station and automatic station	6.33	6.73	106.4
Routine station, automatic station, and EO	7.38	2.86	41.6

^*^Calculated from daily Chl-a and SD

## Discussion

The daily measurements of the automated station decreased uncertainty as compared to the routine sampling station, where sampling frequency was that of the typical Chl-a monitoring used in the WFD. The number of water samples taken during the annual WFD monitoring period in Lake Pyhäjärvi in 2019 was eight compared to the mean frequency of EU countries which was about six (Papathanaopoulou et al., 2019). The DFS results of the whole study area, however, showed the importance of spatial data in improving the confidence of monitoring and status estimation.

Satellites with sensors developed for water quality applications provide images daily (with two Sentinel-3 OLCI instruments, 300-m resolution), and high-resolution images are available with a 2–3-day interval (with two Sentinel-2 MSI instruments, 10–20 m) situated on the same latitude as Lake Pyhäjärvi. Despite occasional cloud cover, the satellite sensors provide more temporal water quality observations than typical WFD station-based sampling. However, a globally valid EO algorithm for lake water quality estimations is not available (e.g. Pahlevan, et al., 2021). As a consequence, regional algorithms and national portals have been developed (e.g. TARKKA portal, https://wwwi4.ymparisto.fi/i4/eng/tarkka).

Several water quality interpretation algorithms are publicly available (e.g. https://earth.esa.int/eogateway/tools/snap) for satellite image processing. Their application to new lakes and regions requires validation and/or calibration of the water quality estimations. EO is a relatively new method to many lake monitoring and reporting authorities. Wider use of EO, e.g. in WFD monitoring, requires promotion and support as indicated in the White Paper of the EOMORES project (Papathanaopoulou et al., 2019). One of its recommendations was to create an EO expert group to harmonize metrics in EU countries and advise member states on the best practices.

Another technique to improve the spatial accuracy of monitoring is to measure transects with flow through sensors from a moving boat (Kallio et al., 2015; Koponen et al., 2007; Lindfors et al., 2005; Scheinin & Asmala, 2020). These measurements can be made at a speed of 15–20 knots, i.e. 37 km at maximum can be covered in an hour, with a typical spatial resolution of 10’s metres. The required devices for transect measurements are compact, and the boats used for routine water sampling can be easily equipped with them. A few transects at a time could be measured each time a lake is visited for routine water sampling. Transect measurements can be easily added to DFS as it accepts any type of observations.

DFS of multisource data is based on parameters, which enable estimation of water quality and Chl-a-based status estimation of a desired spatiotemporal scale. These parameters include, e.g. the initial mean and uncertainty of the state estimates. Excluding these parameters, the other system parameters are hard to optimize using the available data. First, the amount of available data is not very large. Without regularizing the optimization, the parameter optimization would lead to overfitting and poor extrapolation and interpretability of the results. Secondly, making a prediction with a single parameter configuration takes more than an hour. This makes optimizing the parameters with gradient-based methods inherently slow. The chosen parameters are well aligned with the previous application of DFS (Gunia et al., 2022). However, some parameters, such as correlation length, can differ substantially between sites that are different in terms of size, water depth, location, or other properties (e.g. Fang et al., 2019). The authors changed the parameters by factors of approx. 0.5–1.5 to see if there was a meaningful impact on the simulation results but detected none. If EO data (which is the most complete spatially) are available, the computed estimates are very robust to deviations in the model parameters. However, in the absence of EO data, model drift was found to markedly affect the results. In addition, the correlation length computed from the EO measurements considerably affects the estimates in the absence of EO data. In such cases, special care should be taken when selecting these parameter values.

The DFS interpolation is unable to consider dynamic processes such as a rise in Chl-a concentration due to phytoplankton blooms if observations are lacking. One solution to better estimate the temporal variation is to apply the data assimilation technique, in which a hydrodynamic water quality model is utilized to simulate the variable for periods without observations. Measurements are used to improve model simulations whenever available. Assimilation of the measurements is often based on, e.g. Kalman filtering, and measurement and model errors can be taken into account. Data assimilation has been applied particularly to eutrophication and algal bloom modelling (see the review by Cho et al., 2020).

Conventionally, sampling location, frequency, statistical survey design, and sampling protocols are designed to minimize the uncertainty of lake monitoring programs. Here, novel analysis and interpolation methods provided the information necessary to estimate and illustrate spatiotemporal variability of Chl-a and corresponding uncertainty in the study area and to guide the spatial and temporal allocation of multisource monitoring resources.

## Conclusions

EO provides spatially extensive water quality observations with limited frequency, whereas automated stations provide temporally frequent data from few locations. The information value of these observations has rarely been evaluated in comparison to conventional monitoring, which is mainly based on infrequent water sampling at lake deeps.

The spatiotemporal interpolation of multisource Chl-a observations in our research made it possible to compare the information value of various observation types in estimating the ecological status of the research area and quantified the uncertainty of the information from Lake Pyhäjärvi. In addition, the contribution of different measurement types to the uncertainty of interpolation estimates was studied. The method described can be applied to any water quality variable in a lake or coastal environment, and the system accepts any type of observation.

We showed that satellite observations substantially reduced the uncertainty of the estimated interpolation throughout the study area. The deployment of spatial Chl-a observations is recommended either as EO observations or flow-through fluorometer measurements from a moving boat as supplementary material to conventional monitoring. The interpolation method presented can be used to allocate multisource monitoring resources in space and time to reduce uncertainties in the status estimation of a lake.

## Data Availability

The results/data/figures in this manuscript have not been published elsewhere, nor are they under consideration (from you or one of your contributing authors) by another publisher.
